# Single Cell Mathematical Model Successfully Replicates Key Features of GBM: Go-Or-Grow Is Not Necessary

**DOI:** 10.1371/journal.pone.0169434

**Published:** 2017-01-03

**Authors:** Elizabeth Scribner, Hassan M. Fathallah-Shaykh

**Affiliations:** 1 The University of Alabama, Birmingham, Department of Mathematics, Birmingham, Alabama, United States of America; 2 The University of Alabama, Birmingham, Department of Neurology, Birmingham, Alabama, United States of America; Wake Forest University School of Medicine, UNITED STATES

## Abstract

Glioblastoma (GBM) is a malignant brain tumor that continues to be associated with neurological morbidity and poor survival times. Brain invasion is a fundamental property of malignant glioma cells. The Go-or-Grow (GoG) phenotype proposes that cancer cell motility and proliferation are mutually exclusive. Here, we construct and apply a single glioma cell mathematical model that includes motility and angiogenesis and lacks the GoG phenotype. Simulations replicate key features of GBM including its multilayer structure (*i.e.*edema, enhancement, and necrosis), its progression patterns associated with bevacizumab treatment, and replicate the survival times of GBM treated or untreated with bevacizumab. These results suggest that the GoG phenotype is not a necessary property for the formation of the multilayer structure, recurrence patterns, and the poor survival times of patients diagnosed with GBM.

## 1 Introduction

Glioblastoma is a malignant brain tumor that causes high morbidity and poor survival times. Brain invasion, motility, and rapid proliferation are characteristic features of malignant glioma cells. The hypothesis that cancer cell motility and proliferation are mutually exclusive first emerged in the work of Giese *et al.*, who studied cell migratory and proliferative response to extracellular matrix proteins in vitro. Their findings suggested a dichotomy between the two behaviors [1]. Hatzikirou *et al.* later coined the phrase “Go-or-Grow” (GoG) to describe this dichotomy, suggesting that this phenomenon best explains the transition of malignant tumors to the invasive phenotypes in the presence of hypoxia. To test this hypothesis, they apply a lattice-gas cellular automata model, which we hereafter refer to as the Dresden model [2]. Finally, Dhruv *et al.* recently reported that the coordinated suppression and activation of certain transcription factors may explain the shift in glioma cells from the proliferating to migrating phenotype [3].

In previous work, we have developed a mathematical model of Glioblastoma multiforme (GBM), which not only replicates the known multi-layer structure of GBM, *i.e.*necrosis, enhancing ring and edema, but also reproduces specific tumor progression patterns and their associated average patient survival times [4, 5]. This model incorporates the GoG phenotype by including two specific cell types, Invasive (I) cells and Proliferative (P) cells, which can switch back and forth between one phenotype or another depending on the nutrient environment in the brain. As suggested by their names, I cells move throughout the brain but do not divide, and P cells divide but do not move; under the assumption of the GoG phenotype, a single tumor cell will never move and divide at the same time. The equations in [4, 5] also include an angiogenic term.

Our previous work uncovered motility as a possible predictor of GBM progression patterns. Unique to our model is a hypoxia-driven motility term, which is different from diffusion, or concentration-driven dispersion, in that it allows tumor cells to react to low-oxygen environments with increased motility. Through titrations of these motility terms, we have identified three distinct patterns of motility in GBM tumors: (1) highly dispersive, (2) moderately dispersive, and (3) hypoxia driven. These motility phenotypes are associated with different survival times as well as the way a GBM reacts to anti-angiogenic (AA) treatment. The math model includes parameters that model fundamental properties of the tumor including the motility rate, which we found to be a distinguishing factor in the progression types (PT) [6]. We have identified these three types as Expanding FLAIR, Expanding FLAIR + Necrosis, and Expanding Necrosis.

Baker *et al.* recently developed an agent-based model of GBM, simulating perivascular brain tumor growth and invasion, that does not include the GoG phenotype [7]. In their model, which simulates the displacement of each cell, cancer cells in contact with blood vessels move slower due to adhesion and divide at a fixed mitotic rate. Experimental data from Baker *et al.* supports the idea that some glioma cells retain the ability to both divide and migrate on blood vessels [7]. In an effort to better understand the GoG phenotype, here, we develop a new mathematical model at the scale of MRI which includes all the features of our two-cell model (angiogenesis, hypoxia-driven motility, concentration-driven motility, and proliferation) with the exception of the GoG phenotype. Rather than include two specific cell types, one that divides and one that moves, our new model includes one cell type, Glioma cells (G), which can both move and divide at the same time.

In this investigation, our goals were to use the new single glioma cell mathematical model to (1) replicate the multilayer structure of GBM for untreated tumors in all three motility phenotypes of GBM (highly dispersive, moderately dispersive and hypoxia-driven), (2) replicate the three progression patterns associated with bevacizumab-treated tumors (Expanding FLAIR, Expanding FLAIR + Necrosis, Expanding Necrosis), and (3) replicate the survival times associated with these three progression patterns. Our results suggest that the GoG phenotype may not, in fact, be necessary.

In the sections that follow, we first present the equations governing the single glioma cell model, explaining the key differences between this model and our original two-cell model. We then show the results of simulations using our new model, including a simulated clinical trial. We conclude by proposing that the GoG phenotype is not an absolute necessity for the formation of the multilayer structure of GBM and its recurrence patterns.

## 2 Materials and Methods

### 2.1 Mathematical Model

**Fig 1:**
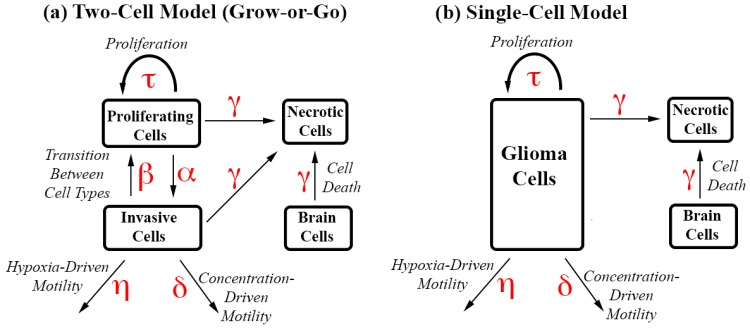
Interactive Cell Type Diagrams with Parameters. Parameters driving different types of cell movements or transitions are shown in red. (a) The Two-Cell Model, which incorporates the GoG phenotype, includes two distinct cell types. Invasive cells can only move or die, and proliferating cells can only proliferate or die. The cells switch phenotypes in the presence of hypoxia (*P* → *I*) and normoxia (*I*→P). (b) The Single-Cell Model has a single glioma cell phenotype, which can proliferate, move or die.

The authors have recently reported a system of partial differential equations (PDE) that models GBM at the scale of MRI; the equations model replication, brain invasion, angiogenesis, and hypoxia [4, 5]. Here, we modify the system of equations to eliminate the GoG phenotype, thus reducing the system to a single glioma cell model; the equations are shown in Table 1, and the parameter values and units are displayed in Table 2. In both models (see Fig 1 and Tables 1 and 3), the brain tissue is taken to be homogenous so that the rate of diffusion is constant throughout the brain. The new system of equations includes a single PDE for glioma cells (see Table 1 and Fig 1b). These cells can multiply and migrate using two modes of motility: concentration-driven (passive transport) and hypoxia-driven (active transport) motility. A description of the major differences in these two modes of motility can be found in [4, 5]. Most notably, passive diffusion is driven by glioma cell concentration, and active transport is driven by hypoxia, or low nutrient conditions, which varies inversely with total cell concentration. Passive diffusion is blind to hypoxia, whereas active transport causes cancer cells to move in bulk away from necrosis and into healthy brain tissue.

**Table 1:** The system of equations for the Single Glioma Cell Model.

$$\text{Glioma Cells}:\partial_{t}G=\underset{\text{Passive diffusion of G cells}}{\underset{︸}{\delta \nabla \cdot (D\nabla G)}}-\underset{\text{Active transport of G cells}}{\underset{︸}{\eta \nabla \cdot (G\nabla B)}}+\underset{\text{Net production of G cells}}{\underset{︸}{MG}}-\underset{\text{Necrosis of G cells}}{\underset{︸}{\gamma FG}}$$ (1)
$$\text{Brain Cells}:\partial_{t}B=\underset{\text{Necrosis of brain cells}}{\underset{︸}{-\gamma FB}}$$ (2)
$$\text{Necrotic Cells}:\partial_{t}N=\underset{\text{Conversion of G and B to necrotic cells}}{\underset{︸}{\gamma F(B+G)}}$$ (3)
Assumptions regarding angiogenesis, hypoxia, mitosis, and necrosis: Total cell concentration:C=G+B+N (4)
$$\text{Mitotic Rate}:M=\tau \left(\frac{\tanh(100\left(C_{ltm}-C\right))}{2}\right)$$ (5)
$$\text{Rate of necrosis}:\gamma F=\gamma \left(\frac{1+G}{B+0.01}\right)\left(\frac{1-\tanh(100\left(C_{ltm}-C\right))}{2}\right)$$ (6)
$\text{Necrotic threshold}:C_{ltm}=\sigma \left[\text{log}\left(1+G\right)\right]+\Omega ,$ (7) where *σ* = 1.0 to simulate angiogenesis, and *σ* = 0 to simulate anti-angiogenesis.

A description of the parameters and their values/units may be found in Table 2.

**Table 2:** Parameters of the Single-Cell and GoG Models.

Description	Symbol	Single Glioma Cell Model	GoG Model
Diffusion rate	*δ*	[0, 1 × 10^−3^]*mm*^2^/*hr*	[8 × 10^−7^, 4 × 10^−3^]*mm*^2^/*hr*
Active transport rate	*η*	[1.4 × 10^−4^, 1.4 × 10^−3^]*mm*/*hr*	[1.4 × 10^−4^, 1.4 × 10^−3^]*mm*/*hr*
Rate of conversion of P to I cells	*α*	**n/a**	1.01/hr
Rate of conversion of I to P cells	*β*	**n/a**	1.0/hr
Mitotic rate (max)	*τ*	0.25/hr	0.35/hr
Necrotic rate	*γ*	0.1/*hr*	0.085/*hr*
Angiogenic rate	*σ*	0.8	1.5
Initial threshold	Ω	1.1	1.1
Fixed difference: *C*_ltm_ − *C*_hyp_	Φ	**n/a**	0.1

n/a: not applicable.

**Table 3:** The system of equations for the GoG Model.

$$\text{Proliferative Cells}:\partial_{t}P=\underset{\text{Net production of P cells}}{\underset{︸}{MP}}-\underset{\text{Conversion of P cells to I during hypoxia}}{\underset{︸}{\alpha HP}}+\underset{\text{Conversion of I cells to P during normoxia}}{\underset{︸}{\beta (1-H)I}}-\underset{\text{Necrosis of P cells}}{\underset{︸}{\gamma FP}}$$ (8)
$$\text{Invasive Cells}:\partial_{t}I=\underset{\text{Passive diffusion of I cells}}{\underset{︸}{\delta \nabla \cdot (D\nabla I)}}-\underset{\text{Active transport of I cells}}{\underset{︸}{\eta \nabla \cdot (I\nabla B)}}+\underset{\text{Conversion of P cells to I during hypoxia}}{\underset{︸}{\alpha HP}}-\underset{\text{Conversion of I cells to P during normoxia}}{\underset{︸}{\beta (1-H)I}}-\underset{\text{Necrosis of I cells}}{\underset{︸}{\gamma FI}}$$ (9)
$$\text{Brain Cells}:\partial_{t}B=\underset{\text{Necrosis of brain cells}}{\underset{︸}{-\gamma FB}}$$ (10)
$$\text{Necrotic Cells}:\partial_{t}N=\underset{\text{Conversion of P, I, and B to necrotic cells}}{\underset{︸}{\gamma F(B+I+P)}}$$ (11)
Assumptions regarding angiogenesis, hypoxia, mitosis, and necrosis: Total cell concentration:C=P+I+B+N (12)
$$\text{Measure of Local Hypoxia for P/I Conversion}:H=\left(\frac{1-\tanh(100\left(C_{hyp}-C\right))}{2}\right)$$ (13)
$$\text{Mitotic Rate}:M\left(H\right)=\tau \left(\frac{\tanh(100\left(C_{hyp}-C\right))}{2}\right)$$ (14)
$$\text{Rate of necrosis}:\gamma F=\gamma \left(\frac{P+I+1}{B+0.01}\right)\left(\frac{1-\tanh(100\left(C_{ltm}-C\right))}{2}\right)$$ (15)
$\text{Hypoxic threshold}:C_{hyp}=\sigma \left[\text{log}\left(1+P\right)\right]+\Omega ,$ (16) where *σ* = 1.5 to simulate angiogenesis, and *σ* = 0 to simulate anti-angiogenesis.
$$\text{Necrotic threshold}:C_{ltm}=C_{hyp}+\Phi$$ (17)

A description of the parameters and their values/units may be found in Table 2.

By reducing the two glioma cell model to a single glioma cell model, we were able to eliminate two parameters: the rate of transition from P cells to I cells during hypoxia and the rate of transition from I cells to P cells during normoxia. All other parameter values used in the new model were either identical or within a similar range as those values used in the previous GoG model (Table 2). Fig 1 shows an interactive cell diagram comparing the two models.

**Fig 2:**
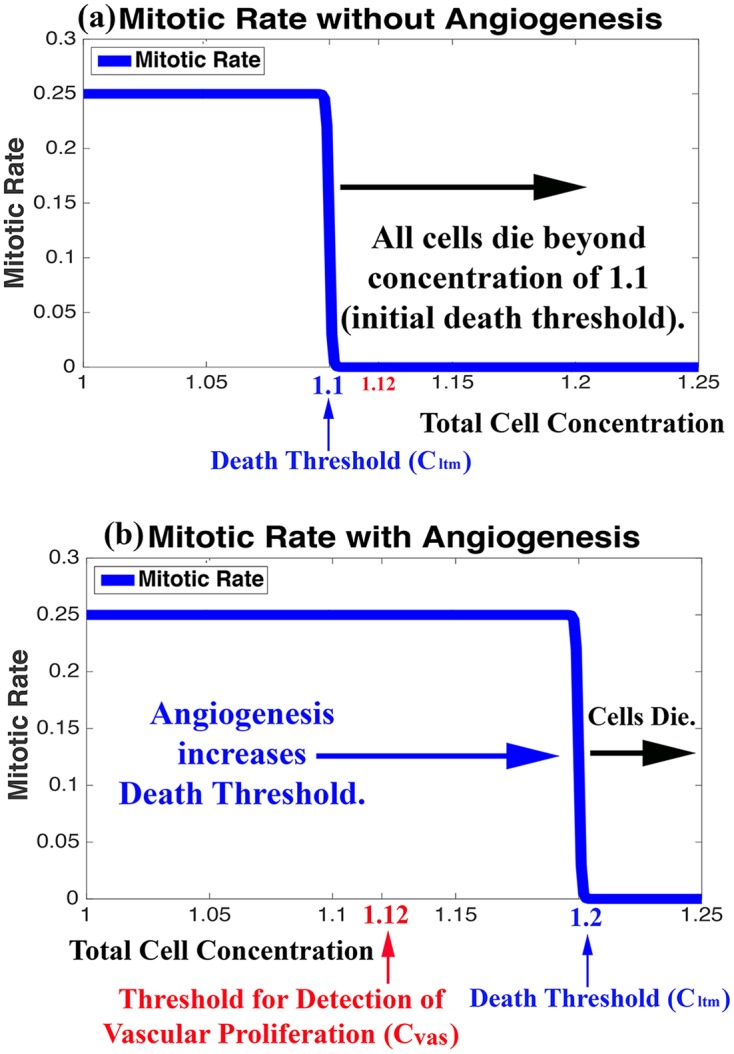
Influence of Angiogenesis on Cell Mitosis and Death. Plots of the mitotic rates of glioma cells in the Single-Cell Model as a function of total cell concentration in the absence (a) and presence (b) of angiogenesis. Mitotic rates drop dramatically and cells begin dying when the total cell concentration reaches the Death Threshold (*C*_*ltm*_), shown in blue. Angiogenesis increases the Death Threshold as a logarithmic function of growing glioma cells, thereby allowing cell concentrations to exceed the Threshold for the Detection of Vascular Proliferation (*C*_*vas*_), shown in red.

The system of equations for the two-cell GoG model is also reprinted below (Table 3). Most notably, our previous model includes two thresholds, one for hypoxia (*C*_*hyp*_) and one for death (*C*_*ltm*_). These thresholds are functions of total cell concentration. The difference in the two thresholds (*C*_*ltm*_ − *C*_*hyp*_ = Φ) created a transition period for the switch of P cells to I cells, and vise versa. Because the single-cell model no longer necessitates this transition, we eliminated the hypoxic threshold and kept just one threshold, (*C*_*ltm*_), which marks the onset of cell death when cancer cell populations reach critical levels. As in our previous model, an angiogenic term can increase the death threshold as a logarithmic function of glioma cells at a fixed rate *σ*, which likely varies from patient to patient. The plots in Fig 2 illustrate the effect of angiogenesis on the death threshold.

### 2.2 Numerical Methods

The original two-cell mathematical model is discussed in detail in [4, 5]. Specific parameter choices, including those differentiating the three motility phenotypes, are also detailed in Table 2 and [4, 5]. These papers also review the numerical methods used to solve the system of equations, which is identical to the methods used to simulate results for the new single glioma cell model. The computations were performed at the Alabama Supercomputer Authority (www.asc.edu).

### 2.3 The Necrotic and Angiogenic Tumor Threshold

The initial brain concentration in our model is 1.0. The initial necrotic threshold, *C*_*ltm*_, is 1.1, which means the tumor mass has reached ten percent of the initial brain concentration, the maximum amount of additional tissue the brain can sustain in our model without further vascularization. Without angiogenesis, the tumor and brain tissue will begin to die once the total cell concentration reaches the initial necrotic threshold. The tumor cells also stop dividing at this point. With angiogenesis, *C*_*ltm*_ begins at the initial hypoxia threshold and increases as a function of glioma cells (G) at a fixed angiogenic rate (per hour), which may vary from tumor to tumor. When the total cell concentration reaches *C*_*ltm*_, division slows and cells begin to die.

Without angiogenesis, a primary characteristic of GBM, tumor cells cannot exceed a concentration of 10% of the brain (*i.e.* total cell concentration cannot exceed the initial necrotic threshold of 1.1). Hence, one of our two methods of detection for GBM is the presence of total cell concentrations above the threshold for the detection of vascular proliferation, *C*_*vas*_. This threshold is set at 1.12, meaning local vascularization has allowed tumors cells to reach a concentration of 12% or more of the brain, which exceeds the initial death threshold by 20%. The plots in Fig 2 show (a) the initial (in the absence of angiogenesis) rate of mitosis and onset of cell death as a function of total cell concentration and *C*_*ltm*_ and (b) the effect of tumor angiogenesis on this rate and death threshold, which allows total cell concentrations to exceed *C*_*vas*_.

### 2.4 Definitions of FLAIR, Vascular Proliferation, and Necrosis

Note that a single pixel is approximately 3 *mm*^2^, which is at the resolution of MRI. The following describes definitions for Angiogenic Tumor, Necrosis, and Fluid Attenuated Inversion Recovery (FLAIR) in our simulations:

1. **FLAIR (*C* > 1.003)**: Our definition of FLAIR is meant to capture any area of the brain that has been invaded by a small concentration of cancer cells. We assume that cell concentrations above 1.003, that is when tumor cells exceed 0.3 percent of the initial brain concentration, generate high signal in FLAIR MRI sequences.
2. **Vascular Proliferation (*C* > 1.12)**: Vascular Proliferation is defined as any area (or pixel) of the brain where the total concentration of cells exceeds the threshold for detection of Vascular Proliferation (*C*_*vas*_) of 1.12. We assume that at this point, the tumor cell density causes a disruption in and increased permeability of the blood-brain barrier, resulting in contrast enhancement on MRI.
3. **Radiological Necrosis (*B* < 0.30)**: Radiological necrosis (that which can be detected at the resolution of an MRI) is defined as any area (or pixel) of the brain where 70% or more of the brain cells in that area are dead.
4. **Pathological Necrosis (*B* < 0.9995)**: Pathological necrosis (that which can be detected under a microscope following tumor resection) is defined as any area (or pixel) of the brain where 0.05% or more of the brain cells in that area are dead.

### 2.5 Simulated Patient Diagnosis and Death: Clinical Trials

In this investigation, we monitor tumor growth, FLAIR, gadolinium enhancement and necrosis; the overall survival time is taken to be the difference in the time of death and the time of diagnosis. We define the time of diagnosis to be the time in the simulation when either (1) the area of tumor with vascular proliferation or (2) the area of radiological necrosis reaches a certain critical threshold (see Table 4). Likewise, the time of death is defined as the time when the area of (1) tumor with vascular proliferation, (2) FLAIR, or (3) radiological necrosis in the brain reaches a critical size. As in the time of diagnosis, the time of death is triggered when the first of these three thresholds is reached.

**Table 4:** Titrations of Diagnostic/Death Criteria for Simulated Clinical Trials.

Trial Group	Group Size	Motility Phenotype	Diagnostic/Treatment Criteria	Death Criteria	Median Survival Time
**Highly Dispersive + Angiogenesis**	25	CoD: High HD: High	% Vascular Proliferation: {1.0, 1.5, 2.0, 2.5, 3.0}	% FLAIR: {62, 63, 64, 65, 66}	14.5 mo
**Moderately Dispersive + Angiogenesis**	25	CoD: Moderate HD: Hig	% Vascular Proliferation: {1.0, 1.5, 2.0, 2.5, 3.0}	% Radiological Necrosis: {3.4, 3.8, 4.2, 4.6, 4.8}	5.5 mo
**HD +Angiogenesis**	25	CoD: Low HD: High	% Vascular Proliferation: {1.0, 1.5, 2.0, 2.5, 3.0} % Radiological Necrosis {0.5, 0.75, 1, 1.25, 1.5}	% Radiological Necrosis: {3.4, 3.8, 4.2, 4.6, 4.8}	6.6 mo
**Control (Untreated)**	25	CoD: High HD: High	% Vascular Proliferation: {1.0, 1.5, 2.0, 2.5, 3.0}	% Vascular Proliferation: {15, 17, 19, 21, 23}	3.2 mo
	25	CoD: Moderate HD: High	% Vascular Proliferation: {1.0, 1.5, 2.0, 2.5, 3.0}	% Vascular Proliferation: {15, 17, 19, 21, 23}	
	25	CoD: Low HD: High	% Vascular Proliferation: {1.0, 1.5, 2.0, 2.5, 3.0} % Radiological Necrosis {0.5, 0.75, 1, 1.25, 1.5}	% Radiological Necrosis: {3.4, 3.8, 4.2, 4.6, 4.8}	

CoD: Concentration-Driven. HD: Hypoxia-Driven.

As in our previous work with the GoG model, we use the single-cell model to simulate clinical trials with “patients” exhibiting the three identified tumor progression patterns: (1) Expanding FLAIR (PP1), (2) Expanding FLAIR + Necrosis (PP2), and (3) Expanding Necrosis (PP3). We also run simulations of untreated tumors in an attempt to replicate the known average survival time of untreated GBM. We selected five titrations of each diagnostic and death criteria to produce a total of 25 “patients” in each of the three tumor progression categories. Once one of the five diagnostic criteria was reached, we simulated anti-angiogenic tumor treatment by setting the angiogenic parameter *σ* to zero. We also let each of these simulations run untreated for a total of 75 untreated tumor simulations. Table 4 displays the titrations used to simulated death and diagnosis in these in silico clinical trials.

## 3 Results

In the absence of the GoG phenotype, the single-cell mathematical model was designed to replicate: 1) the multilayer structure of GBM in untreated tumors (vascular proliferation along with a necrotic core and a proliferating cancer cell ring), 2) the three tumor progression types under anti-angiogenic treatment, and 3) the known survival times associated with each progression type. In our previous two-cell model, which incorporates the GoG phenotype, we achieved all three goals by varying motility alone and fixing all other parameters. Specifically, highly dispersive tumors progressed by Expanding FLAIR, moderately dispersive tumors progressed by Expanding FLAIR and Necrosis, and hypoxia-drive tumors progressed by Expanding Necrosis. In this model, we achieve the same results by varying motility alone but without the GoG phenotype. Table 5 summarizes the conclusions of our simulations, while also providing a comparative analysis to the two-cell model and the other mathematical models referenced in Table 6.

**Table 5:** Comparative Analysis of Simulated Results of Different GBM Models.

Model	Multilayer Structure			Progression Patterns			Survival Times			
	Necrosis	Proliferating Ring	FLAIR	PP1	PP2	PP3	PP1	PP2	PP3	Untreated
Swanson-PI	no	no	yes	no	no	no	no	no	no	no
Swanson-PIHNA	yes	yes	yes	no	no	no	no	no	no	no
Dresden	yes	yes	yes	no	no	no	no	no	no	no
Two-Cell	yes	yes	yes	yes	yes	yes	yes	yes	yes	yes
Single-Cell	yes	yes	yes	yes	yes	yes	yes	yes	yes	yes

Our two models (Two-Cell and One-Cell) along with the Dresden and Swanson-PIHNA models were able to successfully replicate the multilayer structure of GBM (proliferation, invasion, necrosis) at the scale of MRI. However, our models are the only two models shown to successfully replicate the three known progression patterns of GBM under anti-angiogenic treatment as well as the known survival times associated with each progression pattern.

**Table 6:** Comparative Analysis of the Features of Different GBM Models.

Model	CoD motility *δ*∇ ⋅ (∇*I*)	HypD motility *η*∇ ⋅ (*I*∇*B*)	GoG	Angiogenesis	Cell Phenotypes (Behavior)	
**Swanson- PI**	yes	no	no	no	glioma cells (proliferative and invasive)	
**Swanson- PIHNA**	yes	no	no	yes	1. normoxic cells (proliferative/diffusive)	2. hypoxic cells (diffusive)
**Dresden**	yes	no	yes	yes	1. mitotic cells (proliferative)	2. migrating cells (invasive)
**Two-Cell Model**	yes	yes	yes	yes	1. mitotic cells (proliferative)	2. migrating cells (invasive)
**Single-Cell Model**	yes	yes	no	yes	glioma cells (proliferative and invasive)	

The table shows a summary of a few well-known mathematical models and their specific differences. The last two columns display the number of cancer cell phenotypes included in each model as well as the associated behavior of each cell phenotype.

### 3.1 Single-Cell model Replicates Multilayer Structure of GBM

**Fig 3:**
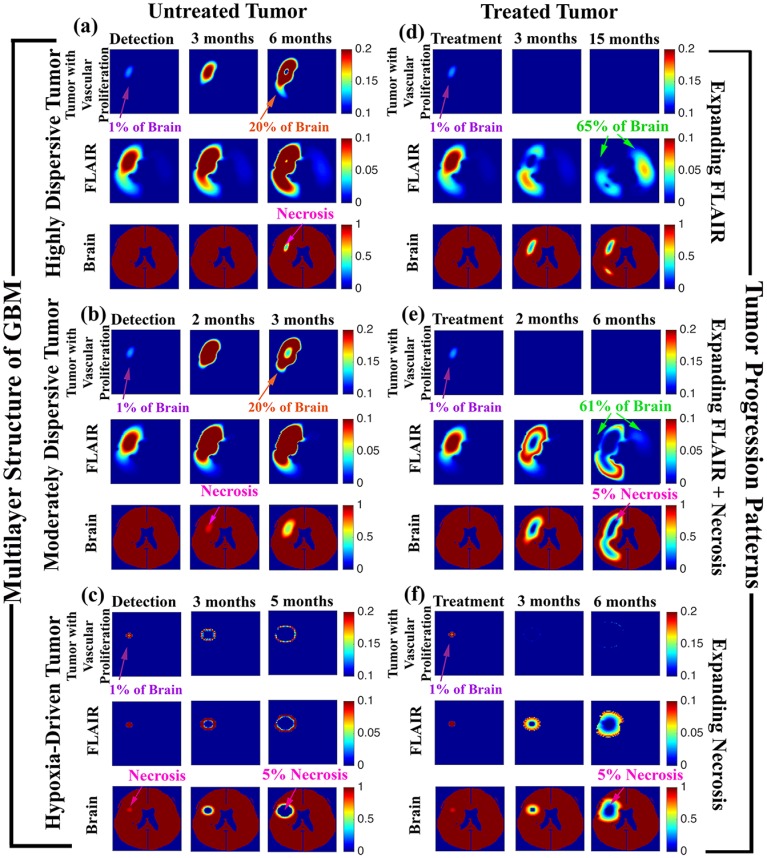
Multilayer Structure and Progression Patterns by Motility Phenotypes. Virtual magnetic resonance imaging of simulations showing the multilayer structure of GBM for the three motility phenotypes: (a) Highly Dispersive, (b) Moderately Dispersive, and (c) Hypoxia Driven. 1% of the brain with detectable vascular proliferation (purple arrows) or radiological necrosis (pink arrows) served as triggers for detection of each GBM. In all three cases, the tumors progressed to the appearance of a necrotic core (pink arrows) surrounded by a proliferating ring (orange arrows). Death was triggered by either 20% tumor mass or 5% radiological necrosis. Treatment of these tumors reproduced progression by Expanding FLAIR (d), Expanding FLAIR + Necrosis (e), and Expanding Necrosis (f). For the treated tumors, the first time shot (treatment) is taken immediately prior to anti-angiogenesis treatment, the second time shot shows the 2-month or 3-month follow-up, and the final time shot displays tumor appearance at the simulated time of death. In progression by Expanding FLAIR (d), treatment effectively eliminates the spread of tumor with vascular proliferation (purple arrows) while low-density tumor cells, or FLAIR (green arrows) continue to invade the brain. Both (e) and (f) also show a reduction in the spread of vascular tumor. However, for moderately dispersive tumors (e), treatment results in progression by Expanding FLAIR (green arrow) + Expanding Necrosis (pink arrow). Treated hypoxia-driven tumors (f) progress by Expanding Necrosis alone (pink arrow), where the area of low-density invasive tumor hovers just beyond the periphery of the necrotic core.

Fig 3(a)–3(c) show the results of simulating an untreated GBM tumor for three choices of motility (Highly Dispersive, Moderately Dispersive, and Hypoxia-Driven). The simulations replicate the multilayer structure of GBM. Note the expansion of vascular proliferation, the development of a proliferating ring, the appearance of necrosis at the core of the tumor, and the spread of FLAIR (low-density tumor) in all three cases.

### 3.2 Single-Cell model Replicates Progression Patterns of Bevacizumab-treated GBM

Our simulations also show that variations in the motility phenotypes can affect tumor response to anti-angiogenic therapy (see Fig 3(d)–3(f)). As in the two-cell model, highly-dispersive tumors (those governed by a high concentration-driven motility parameter) progress by Expanding FLAIR (Fig 3(b)). Treatment effectively reduces the proliferating tumor mass, halting further vascular proliferation; however, the tumor continues to spread at low densities throughout the brain, eventually killing the patient when 65% of the brain has been invaded by FLAIR (green arrow).

For a moderate concentration-driven motility parameter or moderately-dispersive tumor, simulated treatment results in progression by Expanding FLAIR + Expanding Necrosis (see [5]). Fig 3(e) displays this progression pattern. When this moderately-dispersive tumor is treated (first columns), there is a decrease in the proliferating tumor mass, or gadolinium enhancement, but necrosis and FLAIR continue to expand, as evidenced by the growing hole in the brain (pink arrow) and the presence of brain invasion or FLAIR (green arrow) beyond the site of necrosis.

In the presence of very low concentration-driven motility, a high parameter choice for hypoxia-driven motility generates a GBM tumor that progresses by Expanding Necrosis. This treated hypoxia-driven tumor results in an aggressively expanding area of necrosis, as indicated by the pink arrow in Fig 3(f). Note that both the treated and untreated hypoxia-driven tumor simulations (Fig 3c and 3f), glioma cells remain in close proximity to the site of necrosis, which is consistent with the magnetic resonance imaging indicators distinguishing the Expanding Necrosis progression pattern from the Expanding FLAIR + Necrosis progression pattern [5, 6].

### 3.3 Single-Cell model Replicates Survival Times of GBM

**Fig 4:**
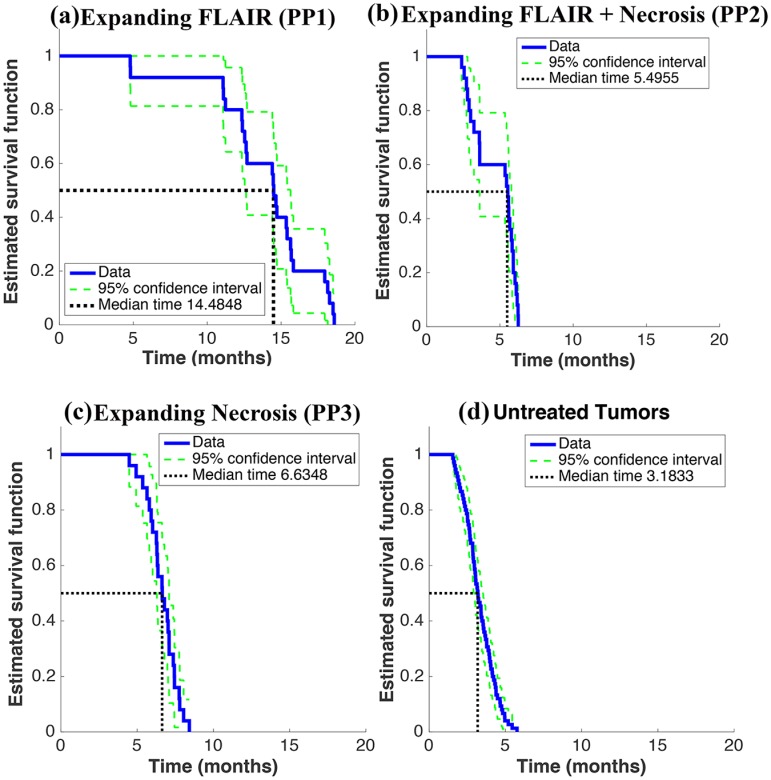
Results of Simulated Clinical Trial. (a)—(d) display a Kaplan-Meier analysis of the overall survival of the four tumor groups in the computational trial. There are three treatment groups: 25 highly-dispersive tumors showing progression by Expanding FLAIR (a), 25 moderately-dispersive showing progression by Expanding FLAIR + Necrosis (b), 25 hypoxia-driven tumors with progression by Expanding Necrosis (c). The control group (d) includes 75 untreated tumors from all three tumor groups.

Fig 4 displays a Kaplan-Meier analysis of the survival times generated by the simulated clinical trial, which includes four distinct ‘patient’ groups: those progressing by Expanding FLAIR (a), Expanding FLAIR + Necrosis (b), Expanding Necrosis (c), and untreated patients (d). The median survival times for each group were close to those found in [6] and [4]. For our computational trial, we found Log-Rank *p* < 0.0001 for comparisons between all trial groups (PP1 vs. PP2, PP1 vs. PP3, PP1 vs. Untreated, etc.). Nowosielski et al. similarly found significant differences in the posttreatment overall survival (Log-Rank p = 0.001) of the highly-dispersive tumor (T2-diffuse or progression by FLAIR), the hypoxia-driven tumor (T2-Circumscribed or progression by Necrosis), and the untreated group (primary nonresponders) [6].

## 4 Discussion

Table 6 lists a few well-known mathematical models and summarizes their specific differences; only some incorporate the GoG phenotype. The Swanson-PI (proliferation-invasion) model was among the first models of GBM to appear in the literature and includes a single cell phenotype that can both proliferate and diffuse throughout the brain [8]; however, this model does not include angiogenesis or hyopxia-driven motility. Of those listed, note that our two-cell model is one of only two models that incorporate the GoG phenotype. The Swanson-PIHNA (proliferation-invasion-hypoxia-necrosis-angiogenesis) model also includes two distinct cell phenotypes (normoxic and hypoxic) [9]. However, unlike the Dresden or our Two-Cell GoG models, both cell phenotypes in the Swanson-PIHNA model have the ability to diffuse throughout the brain, and the normoxic phenotype can both proliferate and diffuse. Our two-cell and single-cell models are the only models that include a hypoxia-driven motility term, which is different from diffusion. Both our models also include an angiogenic term.

In summary, the single-cell model reproduces all the same biological milestones of GBM as the two-cell model (Multilayer Structure, Progression Patterns, and Survival Times), as summarized in Table 5, suggesting that GoG phenotype may not be a necessity in GBM. These results corroborate the findings of Beker *et al.* [7]. We conclude by proposing the hypothesis that the presence or absence of the GoG phenotype may be cell-specific and/or a function of the local environment.

## References

[1] Giese A, Loo MA, Tran N, Haskett D, Coons SW, Berens ME. Dichotomy of astrocytoma migration and proliferation. Int J Cancer. 1996;67(2):275–282. 10.1002/(SICI)1097-0215(19960717)67:2<275∷AID-IJC20>3.3.CO;2-Y 8760599

[2] Hatzikirou H, Basanta D, Simon M, Schaller K, Deutsch A. ‘Go or grow’: the key to the emergence of invasion in tumour progression? Math Med Biol. 2012;29(1):49–65. 10.1093/imammb/dqq011 20610469

[3] Dhruv HD, McDonough Winslow WS, Armstrong B, Tuncali S, Eschbacher J, Kislin K, et al Reciprocal activation of transcription factors underlies the dichotomy between proliferation and invasion of glioma cells. PLoS ONE. 2013;8(8):e72134 10.1371/journal.pone.0072134 23967279 PMC3744529

[4] Raman F, Scribner E, Saut O, Wenger C, Colin T, Fathallah-Shaykh HM. Computational Trials: Unraveling Motility Phenotypes, Progression Patterns, and Treatment Options for Glioblastoma Multiforme. PLoS ONE. 2016;11(1):e0146617 10.1371/journal.pone.0146617 26756205 PMC4710507

[5] Scribner E, Saut O, Province P, A B, T C, et al Glioblastoma Grows During Anti-Angiogenesis: Model to Clinical Predictions. PLoS ONE. 2014;9(12):e115018 10.1371/journal.pone.0115018 25506702 PMC4266618

[6] Nowosielski M, Wiestler B, Goebel G, Hutterer M, Schlemmer HP, Stockhammer G, et al Progression types after antiangiogenic therapy are related to outcome in recurrent glioblastoma. Neurology. 2014;82(19):1684–1692. 10.1212/WNL.0000000000000402 24727314

[7] Baker GJ, Yadav VN, Motsch S, Koschmann C, Calinescu AA, Mineharu Y, et al Mechanisms of glioma formation: iterative perivascular glioma growth and invasion leads to tumor progression, VEGF-independent vascularization, and resistance to antiangiogenic therapy. Neoplasia. 2014;16(7):543–561. 10.1016/j.neo.2014.06.003 25117977 PMC4198934

[8] Swanson KR, Bridge C, Murray JD, Alvord EC. Virtual and real brain tumors: using mathematical modeling to quantify glioma growth and invasion. Journal of the Neurological Sciences. 2003;216(1):1–10. 10.1016/j.jns.2003.06.001 14607296

[9] Swanson KR, Rockne RC, Claridge J, Chaplain MA, Alvord EC, Anderson AR. Quantifying the role of angiogenesis in malignant progression of gliomas: in silico modeling integrates imaging and histology. Cancer Res. 2011;71(24):7366–7375. 10.1158/0008-5472.CAN-11-1399 21900399 PMC3398690

